# The role of PHIRI in the EHDS2

**DOI:** 10.1093/eurpub/ckac129.369

**Published:** 2022-10-25

**Authors:** E Bernal-Delgado, J Gonzalez-Garcia, P Derycke, N Schutte, P Bogaert, M Saso

**Affiliations:** 1 Data Science for Health Services and Policy Research, Institute for Health Sciences, IACS, Zaragoza, Spain; 2 EU Health Information Systems, Sciensano, Brussels, Belgium

## Abstract

The Population Health Information Research Infrastructure (PHIRI) is setting the foundations for the design, implementation and deployment of a fully operative European research infrastructure on population health research in the next few years. In a nutshell, this effort represents setting up the principles and instruments for the governance of the reuse of data for research purposes in a number of aspects - the legal and ethical foundations for the mobilization of data, including sensitive data; how the different nodes in the infrastructure organize to respond multiple research questions in a federated manner; how to ensure high standards of data quality and data interoperability; and, how technological solutions can preserve a safe use of the data while enabling sound scientific outputs. PHIRI has been challenged to provide real-life solutions to each of these aspects. Acting as a demonstration project, PHIRI is currently providing solutions to researchers that are trying to solve their research questions. Building on this experience, in the future, PHIRI is expected to provide support and services to population health researchers that build their research on the secondary use of health data. Among those services: data discovery; data harmonization services (e.g., discovery and use of common data models and interoperability standards); technological services (e.g., playgrounds with synthetic data, secure process environments); research outputs archival (e.g., maintenance and dissemination of the knowledge base generated in the infrastructure); and, training and capacity building. Interestingly, PHIRI can be seen as a primer for the upcoming European Health Data Space for secondary use (EHDS2). In this workshop will be discussing how to line up PHIRI's vision and role within the EHDS2.

